# Bridging Food Systems and One Health: A key to preventing future pandemics?

**DOI:** 10.1016/j.onehlt.2024.100727

**Published:** 2024-04-10

**Authors:** Marcia Arredondo-Rivera, Zoe Barois, Gustavo Enrique Monti, Johanna Steketee, Annabelle Daburon

**Affiliations:** a Wageningen Economic Research, Wageningen University & Research, Atlas (gebouw 104), Droevendaalsesteeg 4, 6708 PB Wageningen, the Netherlands; b Wageningen Centre for Development Innovation, Wageningen University & Research, Droevendaalsesteeg 1, 6708 PB Wageningen, the Netherlands; c Quantitative Veterinary Epidemiology group, Wageningen University, Droevendaalsesteeg 1 (Campus) Building no. 107, 6702 PB Wageningen, the Netherlands

**Keywords:** Zoonoses, One Health, Food system, Operationalization

## Abstract

Food Systems and One Health are two approaches increasingly known for the holistic perspective they bring when addressing the issues that concern them: food and health. This systematic literature review aims to explore the evidence for using these approaches in a concerted manner to better manage zoonoses. By zoonoses management, we refer to improving the ability to address current zoonoses as well as preventing future ones. A total of 98 scientific articles were screened, of which 29 were considered eligible due to their focus on operationalizing each approach to help address zoonoses, as well as a combination of the two. Most articles implement One Health to prevent zoonoses by guiding stakeholders in concerted and participatory decision-making processes. However, the One Health approach can also be adopted via data modelling. Several articles refer to the monitoring and evaluation process of One Health initiatives to prevent zoonoses and discuss best practices to successfully implement the approach. Contrastingly, only three studies adopt a Food System approach to manage zoonoses, despite the profound connections existing between our food systems and the emergence of zoonotic risks. We conclude that there is a lack of integration between the One Health and Food System approaches to manage zoonoses. We also show that experts call for integration, so that not only human, animal, plant, and environmental health are considered, but also the socio-economic trade-offs when monitoring and developing strategies to manage zoonoses. This can be reversed, enabling zoonotic risks to be addressed when planning for our food systems of tomorrow.

## Highlights

• Literature related to One Health, Food Systems, and zoonoses in combination are extremely scarce.
• A lack of literature persists on using the Food Systems approach to manage zoonoses.
• The One Health approach can address zoonoses through guided decision-making or data modelling.
• Incorporating all One Health domains is crucial for effective implementation.
• Experts advocate for the integration of the One Health and Food System approach to manage zoonoses.

## 1 Introduction

The Human Development Index (HDI) assesses progress in key areas of human development: longevity, education, and living standards. Recent data indicates a consecutive decline in global HDI for 2020 and 2021[1], largely due to the COVID-19 pandemic. Most emerging infectious diseases globally originate from animals [2]. Zoonotic diseases pose significant challenges to society, affecting health, livelihoods, and food security [3]. Historical pandemics like HIV/AIDS, SARS-CoV, MERS-CoV, and SARS-CoV-2 exemplify varying impacts on human, animal, and environmental health [4]. The risk of zoonotic pandemics has escalated over the past decade due to intricate interactions between pathogens and hosts, alongside shifts in climate, socio-cultural dynamics, and economic factors [2].

Agriculture is identified as the primary environmental driver of new disease outbreaks in the World Health Organization (WHO) Manifesto from COVID-19 [5]. Activities performed across food systems (FS) (e.g.: production, slaughtering, retail, consumption) are known to be a risk to human health due to exposure to zoonotic pathogens. The Food Systems approach (FSA) comprehensively understands the interconnectedness of processes such as the production of food, its processing, distribution, preparation, and consumption, and studies them within their socio-economic and environmental context [6,7]. FSA is increasingly used to assess and shape policies and interventions aimed at promoting sustainability. This study confidently acknowledges the interdependence of three primary outcomes of FS: socio-economic, food and nutrition security, and environmental outcomes. The FSA, however, does not adequately represent health, rather it mostly covers the nutritional aspect of health.

Following the COVID-19 pandemic, the One Health approach (OHA) has gained a new momentum. This approach is based on the recognition that human, animal, and ecosystem health are interconnected and aims to sustainably balance all three [8]. The OHA can significantly improve public health by offering an understanding of the intricate relationships between zoonotic disease determinants and applying this knowledge to predict or prevent new outbreaks. Furthermore, the approach offers a holistic framework for effectively organizing preparedness and control measures for new outbreaks and their consequences [9]. While implementing OHA can be challenging, due to the difficulty of coordinating relevant sectors and disciplines to develop joint actions, efforts to support implementation are in place. For example, the quadripartite One Health Joint Plan of Action aims to “promote the health of humans, animals, plants and ecosystems and to prevent and manage risks at the human–animal–plant–environment interface” by providing guidance and tools for the effective implementation of multisectoral approaches [10].

Both the OHA and FSA take a systemic approach to addressing complex challenges. Zoonoses are inherently linked to both human and animal health, and FS. The FSA is commonly used by decision-makers but fails to fully cover the health component. On the other hand, the OHA is holistic in its understanding of health, but is challenged in practical applications of the approach towards zoonoses management.

In acknowledging the challenges faced by both approaches, as well as the potential synergies of their combined implementation to manage zoonoses in the context of FS, our study aims to explore the research question: *Is there evidence from the literature that the OHA and FSA are being used in combination to better manage zoonoses?* The research question is based on two main hypotheses: A) to date, there is little empirical evidence on the integration of the OHA and FSA to manage zoonoses; and B) integrating the OHA and FSA is proposed by experts to improve zoonoses management.

Our study presents new perspectives for researchers and decision-makers to more effectively tackle the zoonoses prevalent today, and to prevent future pandemics.

## 2 Materials and methods

To conduct this literature review, the Preferred Reporting Items for Systematic Reviews and Meta-Analyses (PRISMA) guidelines were followed [11].

### 2.1 Search strategy

The databases Scopus, CAB Abstracts, and Web of Science were thoroughly searched for eligible articles using the fields: title, abstract, and keywords. The search strategy was based on combining different concepts, which we captured by using different search terms as listed in Table 1. For each concept, the selection of individual search terms was based on expert knowledge, in addition to synonyms used in peer-reviewed publications. The Boolean operator ‘OR’ was used to combine synonyms related to a concept, while ‘AND’ was used to combine different concepts. Initially, the concept of zoonosis, and its associated search terms (Concept 3 in Table 1), was combined separately with either the concepts of OH or FS (Concepts 1 and 2 in Table 1), to obtain evidence of either approach used in relation to zoonoses. To test the hypotheses on the evidence of both approaches used in combination, a search string was created by combining the three concepts: FS, OH, and zoonoses. To incorporate the implementation aspect of both approaches towards zoonosis, search terms to capture the contribution to decision-making were used in addition to the combinations described above. Search terms such as “tool”,” toolbox”, “practical”, “implementation”, and “decision support”, were preliminarily tested. However, most results were articles that focused on risk assessment tools for zoonosis or on disease transmission, with little or no mention of a decision-support component. Because the search aimed at targeting tools that would support the decision-making process, the team confidently combined Concepts 4 and 5 in Table 1 to effectively target decision-making and operationalization tools.

**Table 1:** List of search terms, categorized under the concepts of interest, used for the systematic literature review.

Concept 1	Concept 2	Concept 3	Concept 4	Concept 5
One health	Food system	Zoonosis	Decision	Tool
One health	Agrifood system (agrofood system)	Zoonotic risks		Operational
	Agrifood chain	Zoonoses		
	Food value chain	Zoonotic disease		

* Symbols such as “*”, and “#” were used for the different search engines to find a maximum number of articles.

A search was conducted on May 19th, 2023, and the results were stored, screened, and cleaned for duplicates using the EndNote® software. Any duplicates that were not automatically detected were manually removed.

### 2.2 Inclusion and exclusion criteria

Eligible manuscripts were studies published in peer-reviewed journals that reported on the operationalization of either the OHA or FSA to: a) manage zoonoses; or b) implement both approaches to address zoonoses. Articles that solely focused on the impacts of zoonoses or pandemics and which lacked empirical examples were excluded, as well as articles that only mentioned zoonoses but did not actively implement OHA or FSA. Publications written in English, Spanish, and French were considered eligible as they were the native language of one or more of the authors. The retrieved articles were subject to an initial screening of the abstract only, and those that successfully passed this step entered a full-text eligibility review. The eligibility of the selected articles was guided by inclusion/exclusion criteria. To ensure impartiality, we excluded conference abstracts, grey literature, non-peer reviewed publications, and case reports. To prevent the risk of bias, four team members participated in the abstract and full-text review screening. Inclusion/exclusion criteria were discussed at different points in the review process and random checks were performed on included/excluded articles. If any discrepancies arose, the research team members discussed the articles once more and recorded their unanimous agreement. The records are available upon request.

## 3 Results and discussion

### 3.1 Overview of the selected studies

**Fig. 1:**
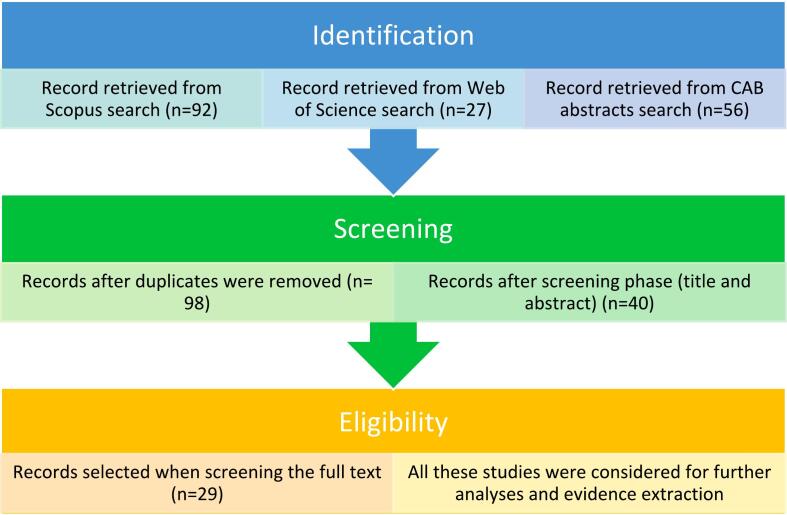
Steps followed during the literature review and number of manuscripts considered at each step (Source: Authors' own elaboration based on PRISMA guidelines [11].

After screening for duplicates, the literature search yielded 98 studies. The article selection process is illustrated in the flow chart based on the PRISMA guidelines (Fig. 1). Titles and abstracts of all studies were screened, of which 40 were included in the eligibility phase. From this, 29 articles met the inclusion criteria and were analysed. Information on how the included articles operationalized the OHA or FSA to address zoonoses was clustered in association with the hypotheses.

### 3.2 General search results

In this section, the results from the various Concepts combinations are introduced. To illustrate the broad evidence linking OH and zoonoses versus the limited literature on the operational aspect of these concepts, a preliminary search using OH and zoonoses concepts was performed. The search yielded 2081 articles in Scopus. However, upon adding the concept of operationalization, the number of articles significantly decreased to 30 (Table 2). After compiling the results from all three search engines and removing duplicates, the search query combining FSA-related terms and zoonoses retrieved 70 articles, and the addition of the operationalization concept to this query retrieved only one article (Table 2). To directly test Hypothesis A, the search combined all three main concepts: OH, FS, and zoonosis. This combination retrieved 13 articles. Upon adding the operationalization terms, each search query yielded one or zero articles.

**Table 2:** Number of articles per search query.

Search query	Total number of articles*	Included**	Excluded
Concept 2 & 3: FSA and zoonoses			
“Food system” OR “Agrifood system” OR “Agrifood chain” OR “Food value chain” AND “zoonosis” OR “zoonotic risks” OR “zoonoses” OR “zoonotic disease”	70	14 [3,[12], [13], [14], [15], [16], [17], [18], [19], [20], [21], [22], [23], [24]]	56

Concept 1, 2, & 3: OHA, FSA, and zoonoses			
“One Health” OR “Onehealth” AND “Food system” OR “Agrifood system” OR “Agrifood chain” OR “Food value chain” AND “zoonosis” OR “zoonotic risks” OR “zoonoses” OR “zoonotic disease”	13	6 [3,12,16,17,19,24]	7

Concept 1, 2, 3, 4, & 5 (OHA, FSA, zoonoses, operationalization terms)			
“One Health” OR “Onehealth” AND “Food system” OR “Agrifood system” OR “Agrifood chain” OR “Food value chain” AND “zoonosis” OR “zoonotic risks” OR “zoonoses” OR “zoonotic disease” AND “decision” AND “tool” OR “operation*”	0	0	0

Concept 1, 2, 4, & 5 (OHA, FSA, & operationalization terms)			
“One Health” OR “Onehealth” AND “Food system” OR “Agrifood system” OR “Agrifood chain” OR “Food value chain” AND “decision” AND “tool” OR “operation*”	1	0	1 [25]

Concept 1, 3, 4, & 5 (OHA, zoonoses, and operationalization terms)			
“One Health” OR “Onehealth” AND “zoonosis” OR “zoonotic risks” OR “zoonoses” OR “zoonotic disease” AND “decision” AND “tool” OR “operation*”	30	19 [[26], [27], [28], [29], [30], [31], [32], [33], [34], [35], [36], [37], [38], [39], [40], [41], [42], [43], [44]]	11

Concept 2, 3, 4, & 5 (FSA, zoonoses, and operationalization terms			
“Food system” OR “Agrifood system” OR “Agrifood chain” OR “Food value chain” AND “zoonosis” OR “zoonotic risks” OR “zoonoses” OR “zoonotic disease” AND “decision” AND “tool” OR “operation*”	1	0	1 [45]

*Includes results from all three databases with duplicates removed from the list. **All articles included in the selection after eligibility phase. When adding up eligible articles from all rows presented here, the number is higher than 29 (as presented in Section 3.1.); this is because some articles were retrieved in more than one search query at the same time.

### 3.3 Managing zoonoses with One Health and Food System approaches

This section presents evidence of OHA or FSA being implemented to address zoonoses.

#### 3.3.1 The One Health approach and zoonoses

The concepts of OH and zoonoses are inherently linked. As our focus is on the practical application of the OHA to manage zoonoses, this section provides a detailed account of its operationalization.

The resulting 19 articles focusing on OHA operationalization to address the management of zoonotic diseases can be divided into two categories: those that describe the approach's operationalization (13 articles), and those that evaluate operationalization processes (7 articles). The studies were conducted in India [[26], [27], [28], [29], [30]], Malta and Serbia [31], Australia [32], Uganda [33], Ethiopia [34], and Canada [35]. Some implemented the OHA to address a range of zoonotic diseases [46], while others focused on single diseases such as brucellosis [31], Lyme disease [47], and Kyasanur Forest Disease (KFD) [26].

##### 3.3.1.1 OHA operationalization to prevent zoonoses

The 13 articles clearly demonstrate how the OHA was put into practice through three key themes: participatory processes, data modelling, and literature reviews alluding to frameworks for OH operationalization (see Table 3).

**Table 3:** Studies on the OHA operationalization to prevent zoonotic diseases.

Reference	Study design	Method of operationalization	Specific disease	Scale	Outcomes
[26]	Cross-sectional	PP⁎: Multi-stakeholder workshop	KFD	National: India	Although co-production in OH is challenging and resource intensive, improved intersectoral collaboration can facilitate successful OH operationalization.
[35]	Cross-sectional	Integrated data source modelling	Lyme disease	Regional: Quebec	Social-behavioural and ecological components are important to predict Lyme disease occurrence at local levels.
[36]	Cross-sectional	PP: decision support tool	No	N/A	This tool is a valuable source of guidance and information to facilitate decision-making between OH risk assessment approaches.
[12]	Literature review	Framework for OH operationalization	No	Global	Open data sharing, open science, & international collaboration are required within an OHA to prevent future pandemics.
[27]	Cross-sectional	Data prediction modelling	KFD	National: India	Co-producing integrated risk maps is an important step in managing zoonotic diseases.
[37]	Cross-sectional	PP: One Health Zoonotic Disease Prioritization tool (OHZDP)	No	All scales	OHZDP offers a transparent and timely process for collaborative zoonoses prioritization tasks.
[33]	Cross-sectional	PP: OHZDP tool	No	National: Uganda	The OHZDP tool can facilitate the development of zoonoses-specific multisectoral disease control and prevention strategies.
[28]	Cross-sectional	PP: OHZDP tool	No	Regional: Haryana state, India	OHZDP can help formulate effective monitoring, prevention, and control strategies for zoonoses in regional settings.
[38]	Cross-sectional	PP: OH Systems Mapping and Analysis Resource Toolkit (SMART)	No	Global	OH-SMART can strengthen OH systems and can foster multi-sectoral collaboration.
[30]	Cross-sectional	PP: OHZDP tool	No	Local: Ahmedabad, India	Prioritizing zoonotic diseases on the local level is essential for developing OH strategies.
[34]	Cross-sectional	PP: OHZDP tool	No	National: Ethiopia	The OHZDP tool can foster multi-sectoral collaboration using quantitative and qualitative methods.
[29]	Case study	PP: adaptation of a sensitivity model & use of systems thinking	No	Local: Ahmedabad, India	Prioritizing zoonotic diseases is essential for developing effective OH strategies
[39]	Literature review	Bio-surveillance framework for OH operationalization	No	Global	Integration of bio-surveillance components is essential to build effective disease management strategies.

⁎ *PP: Participatory process.*

Nine articles used participatory processes to operationalize the OHA [26,[28], [29], [30],^33^,^34^,[36], [37], [38]]. Collaborative workshops were held with key stakeholders from various sectors, including: human and animal health; environment [29,30,38] forestry at local, national, or state levels [26]; government and non-government [33,34]; and food safety [36], to implement OHA as a basis of their zoonotic diseases prevention strategy. Multi-stakeholder processes have been widely acknowledged for their ability to integrate knowledge across different sectors and identify priority data gaps that would otherwise impact zoonoses management [26]. It is important to recognize power dynamics between sectors during the co-production process to ensure that no knowledge contributions are overlooked and that co-production processes retain their legitimacy [26,40]. To successfully operationalize the OHA, it is crucial to form inclusive and collaborative multi-stakeholder partnerships across each domain. Stakeholders related to food safety were involved in one study [36], while environmental specialists [29] and forestry [26] experts were involved in other two. However, it is concerning that the remaining articles only emphasized stakeholder involvement in two of the OHA domains: animal and human health. This highlights a disregard for stakeholders associated with the environmental interface, which is unfortunately a common occurrence in OH research [48,49]. Essack [50] emphasizes that the environment is the most dynamic and confounding sector of OH and is thus a critical component of the OH triad.

Six of the eligible studies implemented the OHA using specific tools: the One Health Zoonotic Disease Prioritization (OHZDP) tool [28,30,33,34,37], and the One Health Systems Mapping and Analysis Resource Toolkit (OH-SMART) [38]. These approaches have shown positive results, laying the foundation for multisectoral collaboration [33] to improve protocols for infectious disease [38] and to help formulate effective surveillance, prevention, and control strategies for zoonotic diseases [28]. The use of these tools embedded in the OHA has highlighted the importance of creating intersectoral linkages that can effectively address emerging zoonotic diseases [34]. Identifying enabling factors at micro-, meso-, and macro levels also appears to be a way forward, while recognizing factors outside the system (e.g., guidelines/policies and community participation) that drive disease emergence [29]. These authors praise the ability of systems thinking to address the underlying causes of pandemics, and to understand the spread of infection and the multiple consequences of pandemics.

Operationalizing the OHA using data modelling was illustrated in two cases [27,35]. These studies included spatial analysis and distribution mapping components for zoonoses, which required different indicators related to topographical, host, land use factors and public health constraints [27], as well as the interaction of social-behavioural and ecological risk factors [35]. The studies emphasize that the use of quantitative methods through variables spanning all OHA domains is critical for its operationalization.

Two reviews were focused on the operationalization of OHA. The first one conceptualized a strategic framework for effective OHA implementation. The framework includes multi-sectoral collaboration at local, regional, and international levels while recognizing a need for the convergence of respective strategies to prevent future pandemic threats [12]. The second review emphasizes the ‘golden opportunity’ for the global health community to implement effective OHA actions through bio-surveillance systems related to human, animal, and plant health [39]. However, adopting the OHA is challenging, due to the need for long-term, multi-stakeholder commitment to a common goal [12,39].

##### 3.3.1.2 Evaluations of OHA operationalization

Seven articles evaluate OHA operationalization to address zoonotic diseases. Five, including two systematic literature reviews [12,41], evaluate OHA implementation to address zoonotic diseases [12,31,32,41,42], while two evaluate tools implemented to address zoonotic diseases in the context of OHA research [40,43] (Table 4).

**Table 4:** Articles evaluating the OHA operationalization process to address zoonoses.

Reference	Study design	Method of evaluation	Specific disease	Scale	Outcomes
[31]	Literature review: comparative, retrospective case study	Applied the Network for Evaluation of One Health Framework to quantitatively analyze OH implementation by scoring operations and infrastructures, e.g., to support sharing and learning, from zero to one (0 = no integration, 1 = strong integration of OH).	Brucellosis	National: Serbia & Malta	Context and timing are key to determine how, when, and why OHA should be applied.
[40]	Review article/ position paper	Review of the writers' experiences with participatory methods in veterinary research projects with a focus on zoonoses and OH projects.	No	N/A	Identifies areas for improvement in the operationalization of participatory epidemiology in veterinary and OH research: using a more qualitative approach for engaging with communities, mapping power structures in working areas, and being aware of people's roles when using participatory methods.
[12]	Literature review	Performed literature survey on history of major pandemics, OH approach, challenges for implementing OH, and frameworks for implementing the OH approach to prevent future outbreaks.	No	Global	Open data sharing, open science, and international collaboration are required within an OHA to prevent/address future pandemics.
[43]	Position paper	Demonstrates lessons learnt from tripartite programmes. Evaluates frameworks which aim to strengthen OH capacity in countries.	No	National: unspecified	Tripartite evaluation frameworks can enable countries to cross map sectoral needs and create a shared vision for multisectoral coordination.
[32]	Opinion paper	Applies philosophical and qualitative methods to map scientific, ethical, and political responses to emerging infectious diseases in Australia.	No	National: Australia	Effective responses to Emerging Infectious Diseases require all socio-political, ethical, and legal implications to be articulated, publicly debated, and resolved in advance.
[41]	Literature review	Systematic literature review on peer reviewed articles in which the OH approach is used to assess programmes and policies related to brucellosis.	Brucellosis	Global/ national	Success of OH programmes is dependent on the willingness of decision-makers and on integration among stakeholders and experts.
[42]	Cross-sectional	Survey among public health and veterinary experts from different Francophone countries on their experience with the OH approach related to zoonotic neglected tropical diseases, followed by two workshops: one on scientific aspects and one on operational aspects.	No	Global: Focus on francophone countries	Difficulties when implementing an OHA relate to connecting health sectors, obtaining dedicated funding, and consistent political support. Advocacy and capacity building are essential for multiplying the benefits of OHA.

The following methods highlighted in the articles provide holistic assessments of OHA implementation: Network for Evaluation of the One Health framework [31]; mapping existing scientific, ethical, and political responses to zoonotic diseases [32]; documenting OHA experiences through surveys [42]; and reviewing literature to: a) evaluate OHA to control human brucellosis [41] and b) discuss potential challenges to implementing OHA [12]. Overall, these studies highlight the importance of an integrated human-animal-ecosystem approach to effectively control zoonoses. While it is recognized as challenging to incorporate all three OHA dimensions in practice, collaboration at multiple levels and among multiple stakeholders remains a critical success factor for implementation. This collaboration can lead to a shared understanding of values and principles among policymakers and public health professionals, which can result in a more effective disease response [41]. Furthermore, due to the diversity of current practices, proper planning is required to create a comprehensive and integrated OHA programme. Buttigieg et al. [31] even conclude that OH strategies should be context-specific, considering time and place, with the right people and using the right infrastructure and operational mechanisms.

A common challenge in operationalization is the development of cross-border capacities and arrangements for rapid detection of emerging health threats. In this regard, open data sharing, open science, and international collaboration are necessary for successful OHA implementation [31]. Assessing OHA operationalization outcomes is also challenging [31], but including qualitative methods to explore the experiences of OHA professionals could be one way to overcome this [42].

Studies evaluating the methods for OHA operationalization [40,43] highlight achieving common ground between visions and collaboration between different disciplines to improve decision-making for zoonoses prevention. Ebata et al. [40] focus on participatory epidemiology as a bottom-up approach to zoonoses control. This method serves to increase the level of engagement among community stakeholders. Contrastingly, de la Roque et al. [43] reviewed multiple toolkits used by the tripartite collaboration: the WHO, the World Organization for Animal Health (WOAH), and the Food and Agriculture Organization (FAO). The focus of this collaboration has been mainly on the human-animal health interface and has lacked the environmental element of OH. With the addition of the United Nations Environment Programme (UNEP) to the consortium, it is expected that environmental aspects will be included in future OH programmes.

#### 3.3.2 FSA and zoonoses

Among the eligible articles, three implement an FSA to address zoonotic diseases [[13], [14], [15]]. Although the authors do not always refer to their approach as being ‘Food Systems’, the articles were included because the authors examined the risks associated with zoonoses using a holistic lens, encompassing various elements of the FS.

One review identified risks for disease emergence by focusing on ‘animal production systems’ and ‘wild harvesting systems’ [13]. This focus enabled the identification of proximal and distal risk factors associated with disease emergence, providing opportunities for disease mitigation and adaptation. Murray et al. [13] highlight that livestock production and wild harvesting systems contribute only a small part of what is needed for a more holistic and proactive strategy to address the risks of disease emergence. Similarly, Shepon et al. [14] construct scenario frameworks to highlight the relative contribution of archetypal FS to zoonotic spillovers and outbreaks. This methodology is then used to suggest policy directions for mitigating risk factors. Despite their focus on the production side of FS, the authors recognize that factors across the entire system collectively influence the risks of zoonotic emergence[14]. Furthermore, by focusing on food-borne zoonotic diseases and food safety, MacKenzie et al. [15] call for concerted efforts by stakeholders across the FS to strengthen public health and implement effective disease risk management strategies.

Despite the important links between FS and zoonoses, the literature covering adoption of FSA to manage zoonoses remains scarce. Although evidence exists on OHA operationalization to manage zoonoses (Section 3.3.1.), it is usually implemented in isolation of the FSA (Section 3.3.2). Therefore, the findings of the literature search are in support of Hypothesis A: to date, there is little empirical evidence on the integration of the OHA and FSA to manage zoonoses.

The following section explores the interest in combining FSA and OHA to better manage zoonoses.

### 3.4 Integrating the Food System and One Health approaches – a pathway towards zoonoses prevention

This section presents arguments in favour of integrating FSA and OHA to manage zoonoses. However, it does so in a theoretical manner, highlighting the lack of empirical evidence of their integration (Table 2). The FSA has several externalities that directly affect human and animal health, some of which relate to the emergence of novel pathogens, foodborne diseases, antimicrobial resistance, and malnutrition [16]. Meanwhile, public health challenges, such as zoonotic disease outbreaks, can severely disrupt the food supply chain [16,17]. The interconnectedness of these adverse outcomes motivates researchers from veterinary, human health, and FS to call for the integration of the OH and FS approaches. This can lead to the development of future interventions that can result in better zoonoses management and FS improvements simultaneously. Patterson et al. [16] argue that when FS operate in an environmentally-sustainable manner, with greater resilience and adaptability, emerging infectious disease events are less likely to occur. Moreover, Bitsoumanou Nkounkou & Temple [18] draw lessons from the COVID-19 crisis in the Congo FS to recommend integrating OHA into agricultural and food sector policies. Their recommendations emerge first from the broad impacts of viral zoonoses, for instance, on human health and development, and second from the risk of zoonotic spillover from animal-human systems due to bushmeat consumption.

Other studies emphasize that integrating knowledge and food security into the design of interventions throughout the FS is critical to preventing future pandemics. For example, Aiyar & Pingali [17] propose market-based approaches to make food safety an attribute of food products. This strategy is expected to increase consumers' willingness to pay for safer food, thereby improving the traceability of disease along value chains. In line with the need for a broader FS perspective when addressing zoonoses, Leahy et al. [3] argue that OH initiatives that only focus on surveillance and disease control in the animal host are insufficient. They suggest the OHA can inspire interventions at multiple FS nodes, which they support by analyzing interventions to help reduce foodborne zoonoses in low- and middle-income countries. The interventions focused on several areas and FS actors; e.g., market infrastructure, sellers and consumers of animal origin foods in traditional markets, and governance of market chains. Boqvist et al. [19] also suggest adopting the OHA when considering food safety and reducing the transmission of zoonotic pathogens in different food products. They argue that disease transmission and antimicrobial resistance phenomena highlight the complexity of FS, and therefore highlight the need to incorporate the OHA to understand how these phenomena occur.

Several studies suggest OHA and FSA should be considered when designing strategies to manage zoonoses, and relevant socio-economic aspects also considered, due to the interconnectedness and dependence of human health and social and economic systems [3,12,19]. They further argue that COVID-19 impacted food supply chains, affecting food security and endangering livelihoods [3,12] Furthermore, trade-offs appear when implementing FS interventions to control disease due to the connection between agricultural production, FS, and associated disease risks [51]. These authors suggest that approaches to disease-risk management should be multi-sectoral, and that intervention designs should begin with an assessment of the economic costs and benefits to both the agricultural and public health sectors. When implementing OH interventions, Buttigieg et al. [31] argue that consideration should be given to the economic consequences of zoonotic diseases. They propose that economic evaluations demonstrating cost savings to governments are crucial for gaining political support for the OHA. Finally, FS decision-makers, in collaboration with OH experts, can play a crucial role in the prevention of emerging infectious diseases [20]. The findings of these studies support Hypothesis B: integration of the OHA and FSA is proposed by experts to improve zoonoses management. Experts suggest integration should take place, for example, to prevent negative outcomes in food supply chains due to zoonotic outbreaks. Some mechanisms proposed for integration include ensuring OH interventions are implemented at various nodes of FS, or embedding OHA into food safety activities throughout food supply chains.

A limitation of our study relates to the inclusion/exclusion criteria, which might limit the number of articles included and, therefore, affect this study's results. Due to our holistic perspective on agrifood, we refrain from addressing specific cases, for instance, of the dairy sector, or wildlife in wet markets. Similarly, the literature search was limited to English search terms, thereby excluding relevant publications written in other languages.

A growing community of decision-makers worldwide is adopting the FSA. Although several health outcomes are captured within the nutrition lens, FSA fails to integrate health aspects that relate to infectious diseases and zoonoses. Social scientists have played an important role in advancing the FSA agenda promoting its adoption by a growing community of decision makers, which was endorsed by the United Nations Food System Summit in 2021. While OH researchers are gaining a new momentum after the COVID-19 pandemic, environmental and social sciences are largely underrepresented in this community of academics [52]. Such a shortcoming may explain the current lack of integration of OHA and FSA to better manage zoonoses, despite their links and the potential benefits of combining them. Bringing social sciences into the OH community through integration with the FSA could open new perspectives to better operationalize OHA, enabling experts to better manage the zoonoses of today to prevent the pandemics of tomorrow. The debate for the global agenda to follow the Sustainable Development Goals (SDGs) agenda will start in the coming years. To date, OH is not included in the SDGs. The scientific community should advocate for the inclusion of OHA and FSA in the future agenda and promote transdisciplinary collaboration for healthier human and animal populations, and consequently a healthier environment.

## 4 Conclusion

The widespread impact of COVID-19 emphasizes the need to develop effective zoonoses management systems; improving the ability to control them, while preventing the emergence of future pandemics. The OHA, which has gained momentum in recent years, serves as a mechanism to work together across human, animal, and environmental health sectors. However, it remains a challenge to operationalize OHA and engage with all stakeholders across the triad of OH sectors, with environmental and social scientists still under-represented in OHA research. Similarly, the FSA is gaining traction with a growing community of decision-makers. Although this approach is based on holistic and systems thinking, it fails to recognize a wide range of relevant health impacts, particularly zoonotic and infectious diseases. By examining OHA and FSA in the context of zoonoses, including their operationalization, this study illustrates the lack of their integration in zoonoses management, despite evident links, which is the first hypothesis (A). This study also illustrates that a growing community of experts are calling for the integration of OHA and FSA approaches to better manage zoonoses, providing support for the second hypothesis (B). In summary, multi-level and multi-stakeholder collaboration is required to successfully implement OHA to manage zoonoses. It is crucial to understand the social and economic impacts of zoonotic diseases and include these when advocating for OHA interventions to control zoonoses. Integrating both approaches, by achieving cooperation between human and animal health, environmental, and FS experts, can lead to interventions that simultaneously improve zoonoses management and FS.

## Funding

This work was supported by Wageningen University & Research through the ERRAZE@WUR programme.

## Declaration of competing interest

The authors have no conflicts of interest to declare. All co-authors have seen and agree with the contents of the manuscript and there is no financial interest to report.

## Data Availability

Data will be made available on request.
